# Evaluation of Immunoassays for the Diagnosis of *Schistosoma japonicum* Infection Using Archived Sera

**DOI:** 10.1371/journal.pntd.0000949

**Published:** 2011-01-18

**Authors:** Jing Xu, Rosanna W. Peeling, Jia-Xu Chen, Xiao-Hua Wu, Zhong-Dao Wu, Shi-Ping Wang, Ting Feng, Shao-Hong Chen, Hao Li, Jia-Gang Guo, Xiao-Nong Zhou

**Affiliations:** 1 National Institute of Parasitic Diseases, Chinese Center for Disease Control and Prevention, Shanghai, People's Republic of China; 2 Diagnostics Research, London School of Hygiene and Tropical Medicine, London, United Kingdom; 3 Zhongshan School of Medicine, Sun Yat-Sen University, Guangzhou, People's Republic of China; 4 Xiangya School of Medicine, Central South University, Changsha, People's Republic of China; Swiss Tropical and Public Health Institute, Switzerland

## Abstract

**Background:**

With a national program initiated recently to reduce transmission of *Schistosoma japonicum* in the People's Republic of China (P.R. China), there is an urgent need for accessible, quality-assured diagnostics for case detection, surveillance, and program monitoring of chemotherapy efficacy and other control interventions in areas of low endemicity. We compared the performance of nine immunodiagnostic tests developed in P.R. China for detection of antibodies against *S. japonicum* and established their priority for further assessment in field settings.

**Methodology/Principal Findings:**

Using the Kato-Katz technique as the reference standard, 240 well-characterized archived serum specimens (100 positive and 140 negative) were evaluated in nine immunological tests developed in P.R. China. The enzyme-linked immunoelectrotransfer blot assay (EITB), which uses an adult worm extract of *S. japonicum*, supplied by the Center of Disease Control and Prevention, USA, was also evaluated. The sensitivity and specificity of each test were determined and the reproducibility of each test was assessed by evaluating operator-to-operator and run-to-run variation. In addition the simplicity of use for the end-user was evaluated. All tests showed good sensitivities ranging from 92.0% (95% confidence interval (CI): 86.7–97.3%) to 98.0% (95% CI: 95.3–100.0%). The test specificities varied from 70.0% (95% CI: 62.4–77.6%) to 97.1% (95% CI: 94.4–99.9%). All tests showed excellent reproducibility with a discordant rate in the range of 0–10.0% for operator-to-operator variation and run-to-run variation. All tests, except one magnetic particle-based enzyme-linked immunosorbent assay, were found to be easy to use, especially the dot immunogold filtration assays.

**Conclusions/Significance:**

Most evaluated tests had acceptable performance characteristics and could make an impact on the schistosomiasis control programs in P.R. China. Three tests with the highest sensitivity, specificity and greatest ease of use, were selected for further evaluation in field settings.

## Author Summary

With the advantages of higher sensitivity and simpler ease of use over stool examination, antibody-detection methods have been integrated into programs for schistosomiasis control in the People's Republic of China after the notable decrease of prevalence and intensity of *Schistosoma japonicum* infection. We compared the performance of nine immunoassays for diagnosis of *S. japonicum* using well-characterized archived serum specimens and prioritized tests for future evaluation. Most tests had acceptable performance characteristics and could have an impact on the control of schistosomiasis. Three tests, including one indirect hemagglutination assay, one enzyme-linked immunosorbent assay and one dot immunogold filtration assay were selected for further assessment in field settings. Our final goal is to have appropriate tools for different stages of schistosomiasis control, such as screening targets for chemotherapy, evaluating the efficacy of schistosomiasis control programs, and monitoring the endemic status of schistosomiasis.

## Introduction

Schistosomiasis, caused by infection with *Schistosoma* spp., remains a public health problem in tropical and subtropical areas of the world. It is currently estimated that 207 million people harbor the parasites and about 779 million people are at risk of being infected with schistosomes [1]. Among three main disease-causing species, namely *Schistosoma mansoni, S. haematobium, and S. japonicum*, the later is the only species endemic in the People's Republic of China (P.R. China). Through more than a half century of effort, the prevalence and intensity of *S. japonicum* infection have decreased significantly resulting in a decrease in the number of infected people, from 11.6 million in the 1950s to about 0.73 million in 2004 [2]–[4]. According to the latest national epidemiological sampling survey, the average prevalence rate was 2.5% in all surveyed endemic areas and 5.1% in the areas where control of schistosomiasis transmission had not been achieved [4]. With the ultimate goal of elimination, a national program was initiated in 2004, with specific objectives to decrease the prevalence of schistosome infection in all endemic counties below 5% in 2008 and 1% in 2015 [5]–[7]. To reach these goals, accurate, simple, and affordable diagnostic tests are needed urgently for case detection, surveillance, and program evaluation, including evaluation of control interventions and verification of elimination of schistosomiasis in areas with very low endemicity.

Demonstration of eggs or miracidia in the feces is considered to be the ‘gold’ standard for the identification of *S. japonicum* infections [8]. The Kato-Katz technique is frequently used in the field because it is quantitative, inexpensive, and easy to use [9], but it can be plagued by decreased sensitivity in areas of low endemicity and particularly in individuals with low worm burdens [10]–[12]. The sensitivity of the Kato-Katz technique can be improved by repeated stool collection and examination but this is more labor intensive and costly [10]–[13]. Nucleic acid amplification techniques based on polymerase chain reaction (PCR) have been reported for *S. japonicum* detection [14]–[17]. However, the methods are not standardized and their sensitivity needs to be verified further. Moreover, the equipment, personnel, and financial investments required are too costly for primary health-care settings in P.R. China.

With the advantages of higher sensitivity and ease of use over stool examination, immunological techniques and methods for antibody detection have attracted scientists' attention. Two national collaborative studies to evaluate the antigens used for detection of antibodies against *S. japonicum* were organized in the 1980's and the results showed crude soluble antigens extracted from parasite eggs performed with the highest sensitivity and specificity [18], [19]. For unknown reasons, no single test evaluated in these studies was further developed or widely adopted in P.R. China. Based on the findings of these previous studies using crude egg antigens, a variety of immunodiagnostic methods such as the circumoval precipitin test (COPT), indirect hemagglutination assay (IHA), enzyme-linked immunosorbent assay (ELISA), dipstick dye immunoassay (DDIA) and dot immunogold filtration assay (DIGFA) have been developed and integrated into national control programs in P.R. China [4,8,20.21]. Although a number of immunodiagnostic kits have been widely used in the field in P.R. China, none had been standardized and licensed by 2008. Furthermore, due to the lack of strict regulatory approval standards, several poorer performing diagnostics are also used. Because so many different tests with differing performance characteristics are being used, it is difficult to interpret the reported data on prevalence of schistosome infection accurately.

In order to pre-qualify available diagnostic tests for *S. japonicum* infection and identify their priorities for future field trials [22], a laboratory-based evaluation for test performance and operational characteristics using panels of well-characterized archived serum specimens from geographically diverse settings of P.R. China was carried out. The study was also extended to include an evaluation of the enzyme-linked immunoelectrotransfer blot assay (EITB) provided by the Center for Disease Control and Prevention (CDC), USA, to determine its utility as an independent reference method for confirmation of cases in low transmission areas.

## Materials and Methods

### Selection of tests for evaluation

Considering the urgent need for quality-assured and approved reagents for diagnosis of schistosome infection in P.R. China, letters of invitation and the study protocol were sent to companies and institutes, and only products that met the following criteria were included for evaluation: (1) tests which have been used in field or clinical settings with reported performance characteristics; (2) tests which could be performed with only minimal training; and (3) products which were in the licensing process. Tests were donated by companies or institutes who were interested in participation in the evaluation, and all test packages were stored according to manufacturers' instructions prior to, and during the evaluation. Well-characterized archived human serum specimens from the serum bank of the China National Reference Center for Schistosomiasis Diagnosis (based at the National Institute of Parasitic Diseases, Chinese Center for Disease Control and Prevention, abbreviated as IPD, China CDC), which were collected in October 2007, were used to evaluate the validity and reproducibility of tests. The examinations were conducted by well trained technicians in a double blind way and operational characteristics of the tests were assessed in one week in May, 2008.

### Collection and handling of specimens

Serum specimens from patients infected with *S. japonicum* were collected in endemic areas in Jiangxi, Anhui and Hubei provinces, P.R. China. *S. japonicum* infection was diagnosed on the basis of the ‘gold’ standard method, demonstration of schistosome eggs in feces with the Kato-Katz technique [9]. Nine slides (41.7 mg feces in each slide) were prepared from three separate stool specimens (three slides for each sample), resulting in evaluation of a total sample weight of 375.3 mg per person. Egg counts reflecting the infection intensity were expressed in eggs per gram of feces (EPG).

Sera were collected from healthy people living in Shandong province who had never traveled to schistosomiasis endemic areas when they underwent routine health check-ups. Serum specimens were also collected from patients with heterologous infections, i.e., hepatitis B, food-borne parasitic diseases (e.g. paragonimiasis, clonorchiasis, and trichinellosis) and soil-transmitted helminthiases (ascariasis, hookworm disease and trichuriasis) in areas free of schistosome infections.

Heterologous infections were diagnosed by stool examinations and/or specific serological tests. Blood samples of 5–10 ml from each donor were collected by venipuncture. Whole blood was kept at 4°C overnight for complete clot retraction. Whole blood was centrifuged and the serum was removed and divided among individual serum storage tubes. Aliquots of serum were frozen at −70°C and linked by a unique numerical code to detailed clinical and parasitological information.

### Preparation of serum panels for evaluation

Assuming a sensitivity and specificity of 90% and 90%, respectively, against the ‘gold’ standard test, and allowing a 6% margin of error, a serum panel sample size of 240 was used for the evaluation. One hundred sera were collected from *S. japonic*um egg-positive persons, which were characterized as 73.0% (73/100) male, with a mean age of 47±13 years. The median infection intensity in patients was 9 EPG and 89.0% had infection intensities below 40 EPG. Fifty serum specimens from healthy persons and 90 specimens from patients with other heterologous diseases (20 cases with hepatitis B, 20 cases with paragonimiasis, and 10 cases each with trichinellosis, clonorchiasis, ascariasis, hookworm disease, and trichuriasis) were also included. Ten aliquots (0.1 ml per aliquot) were prepared per serum specimen and each aliquot was coded with a unique study ID.

For the assessment of reproducibility, panels were prepared with 19 specimens from patients with schistosomiasis and 11 specimens from healthy persons. Each specimen was divided by 40 aliquots (0.1 ml per aliquot) and labeled randomly with unique number.

### Tests for evaluation

A total of nine immunodiagnostic kits developed in P.R. China met the inclusion criteria for evaluation. The antigen for all tests was the crude soluble egg extract of *S. japonicum* and the type of specimen tested in all kits was serum. According to assay type, these kits were classified into three categories: IHA (four tests coded as IHA_AJ, IHA_JX, IHA_XY, and IHA_HB), DIGFA (three tests coded as DIGFA_SH, DIGFA_XC and DIGFA_ZJ), and ELISA (two tests coded as ELISA_SZ and MELISA_BE). Major features of these tests are given in Table 1.

**Table 1:** Major features of the evaluated tests for the diagnosis of *S. japonicum* infection.

Assay code	Assay type	Antigen*	Solid phase	No. samples per run (min. - max.)	Time required per run	Volumes of sample	Extra supplies**	References
IHA_AJ	Indirect hemagglutination	SEA	Red cell from sheep	1–30	<1 h	25 µl	Yes	23,24
IHA_HB	Indirect hemagglutination	SEA	Red cell from sheep	1–30	<1 h	25 µl	Yes	25
IHA_XY	Indirect hemagglutination	SEA	Red cell from sheep	1–30	<1 h	25 µl	Yes	26
IHA_JX	Indirect hemagglutination	SEA	Red cell from humans with “O” type blood	1–30	<1 h	25 µl	Yes	27,28
DIGFA_SH	Dot immunogold filtration assay	SEA	Nitrocellulose membrane	1	<5 min	50 µl	No	29
DIGFA_XC	Dot immunogold filtration assay	SEA	Nitrocellulose membrane	1	<5 min	40 µl	No	30
DIGFA_ZJ	Dot immunogold filtration assay	SEA	Nitrocellulose membrane	1	<5 min	25 µl	No	31
ELISA_SZ	Indirect ELISA	SEA	Microtitre plate	1–96	<2 h	5 µl	Yes	4,32
MELISA_BE	Magnetic particle-based ELISA	SEA	Magnetic particle	1–30	<2 h	5 µl	Yes	33
EITB	Enzyme-linked immunoelectrotransfer blot assay	AWA	Nitrocellulose membrane	1–10	Overnight	3 µl	Yes	34

*SEA  =  soluble egg antigen, AWA  =  adult microsomal antigen.

**Additional equipment of all tests required was consisted of handheld micropipettes and micropipette tips.

The four IHA tests were based on an indicator system consisting of sheep red blood cell or human red blood cell with “O” blood type coated with soluble antigen extracted from *S. japonicum* eggs. The IHA tests were performed as described in previous studies [23]–[28]. The titer in the test sera was recorded as one dilution before that which yielded a clear, sharp dark spot similar to that in the negative control wells. Titers were expressed as reciprocal values. Titers of ≥10 indicated a positive result.

The three DIGFA tests were based on the immunofiltration technique, using anti-human antibody labeled with colloidal gold as the indicator system. The volumes of specimen and reaction liquid were added according to the instructions provided by their manufacturers. For the DIGFA_SH and DIGFA_XC, the appearance of two red dots in the well indicated a positive reaction, and the appearance of a single red dot indicated a negative reaction [29], [30]. For the DIGFA_ZJ, the color of one dot in the well equivalent to or deeper than that of positive control serum was considered a positive result, while only pale or pink background in the well was defined as a negative result [31].

The ELISA_SZ and MELISA_BE were based on the indirect ELISA method but differed significantly in their operational procedures. In the ELISA_SZ, all serum specimens were diluted 1∶100 and transferred into the kit supplied microtiter wells. The incubation procedure, washing steps and detection steps were carried out according to the manufacturer's instructions. Optical density (OD) values were read at 450 nm zeroed by the reagent blank wells. For each run, positive and negative control sera were measured simultaneously. A positive result was defined as an OD value greater than 2.1 times the OD value of the negative control serum provided by the kit, as specified by the manufacturer's instructions [32]. The MELISA_BE test combined indirect ELISA and magnetic particle isolation techniques to identify antibodies against *S. japonicum* [33]. Briefly, serum specimens diluted 1∶200 and fluorescein isothiocyanate (FITC) labeled *S. japonicum* SEA were added to each tube successively. All the washing steps and detection steps were performed as described previously [33]. OD values were read at 550 nm zeroed by the reagent blank wells and the positives were defined as OD values greater than 0.635.

Finally, an EITB supplied by CDC, USA, was also evaluated. The EITB was performed as described in previous reports [34]. A positive result was defined as the appearance of any of three distinct glycoproteins with molecular masses of 18KD, 23KD and 29KD.

### Performance of evaluation

To assess assay performance, each assay was tested with a panel of 240 serum specimens. The testers were blinded to reference standard results and performed the tests independently to avoid comparison of results between kits. Test reproducibility was investigated using a panel of 30 serum specimens. Operator-to-operator reproducibility was compared with two technicians who ran tests with the same 30 sera. Run-to-run variability was investigated using 30 sera that were tested on two different days for each test by the same testers.

Each test was assessed for its operational characteristics by the testing technicians. Tests were scored for clarity of the kit instructions (very clear = 3, clear = 2, not clear = 1), technical complexity (<5 steps and short intervals between steps = 3; 5–10 steps and short intervals between steps = 2; ≥10 steps or long intervals between steps = 1) and ease of interpretation (by eye and easy = 3, by eye but not easy = 2, by machine = 1). The maximum possible score for each index was 3. In addition, a score of 1 was given to the tests that did not require any additional equipment, giving a maximum score of 10. The higher the score, the more suitable the test was considered for use in the field.

### Statistics

All data were processed and analyzed with SPSS statistical software package 13.0 for Windows (SPSS Inc., Chicago, IL, USA). Sensitivities and specificities were calculated relative to the ‘gold’ standard Kato-Katz results that correlated to each serum specimen. The 95% confidence intervals (CIs) were determined for the sensitivity and specificity of each test. Youden's index, expressed as sensitivity + specificity - 1, was used to assess the ability of the test to discriminate true positives and true negatives. Operator-to-operator and run-to-run variability were calculated as the number of tests for which different results were obtained by two independent operators, two different days, divided by the number of specimens tested. Categorical variables between groups were compared by χ^2^ test or Fisher's exact test as appropriate. Significance was assigned at *P*<0.05 for all parameters and was two-sided unless otherwise indicated.

### Ethics statement

The study was approved by the Ethics Committee of IPD, China CDC. The study procedures, potential risks, and benefits were explained to the village leaders. After their consent to perform the study, field workers visited the homes of the selected families where detailed information was provided to all potential participants, and questions were answered. All adult participants and parents/guardians of child participants in a given household provided informed consent. Written confirmation that full information had been provided and individual participation was voluntary (informed consent) was obtained from the head of each participating household or a literary substitute (adult or relative), and this procedure was approved by the aforementioned ethical committee.

Collection of serum specimens was conducted with approval from the Ethics Committee and Academic Board of IPD, China CDC. All personal identifiers and patient information were delinked from the serum specimens.

## Results

The sensitivity of each immunological test using 100 serum specimens collected from *S. japonicum*-infected individuals is detailed in Table 2. The IHA_HB, MELISA_BE and EITB tests performed with the highest sensitivities, 98.0% (98/100; 95% CI: 95.3–100.0%), while the IHA_JX and DIGFA_SH tests showed the lowest sensitivities (92.0%; 92/100; 95% CI: 86.7–97.3%). The nine evaluated tests did not differ from EITB in sensitivity (*P*>0.05). The 47 false negative specimens were mostly from patients with 20 or less EPG.

**Table 2:** Detection of antibodies in *S. japonicum* egg-positive patients (n = 100).

Assay code	No. of true positives	No. of false negatives	No. of EPG* for misdiagnosed	Sensitivity (%) [95% CI]	*P* value against EITB
IHA_AJ	93	7	3, 3, 5, 5, 16, 21, 139	93.0 [88.0–98.0]	0.17
IHA_HB	98	2	5, 21	98.0 [95.3–100.0]	1
IHA_XY	96	4	3, 5, 11, 139	96.0 [92.2–99.8]	0.68
IHA_JX	92	8	3, 3, 5, 11, 19, 21, 27, 139	92.0 [86.7–97.3]	0.1
DIGFA_SH	92	8	3, 5, 5, 5, 8, 11, 11, 37	92.0 [86.7–97.3]	0.1
DIGFA_XC	97	3	11, 24, 37	97.0 [93.7–100.0]	1
DIGFA_ZJ	94	6	3, 5, 5, 5, 11, 37	94.0 [89.3–98.7]	0.28
MELISA_BE	98	2	5, 21	98.0 [95.3–100.0]	1
ELISA_SZ	95	5	3, 3, 5, 8, 37	95.0 [90.7–99.3]	0.45
EITB	98	2	11, 35	98.0 [95.3–100.0]	-

*EPG: egg per gram feces.

The cross-reactivity with heterologous serum specimens and specificity of each evaluated test is listed in Table 3. Cross-reactivity mainly resulted with sera from patients with paragonimiasis (false positive rates: 40.0–60.0%) for all tests except the EITB. Otherwise, false positive rates of each test were very low (0–20%) for patients infected with soil-transmitted helminths, including ascariasis, hookworm disease, and trichuriasis. The highest specificity was observed with the EITB, 97.1% (136/140; 95% CI: 94.4–99.9%). The DIGFA_SH showed the highest specificity (95.1%; 116/122; 95% CI: 91.2–98.9%) among the other nine tests developed in P.R. China, while IHA_XY performed with the lowest specificity (70.0%; 98/140; 95% CI: 62.4–77.6%).

**Table 3:** False positive results of the evaluated immunoassays.

Assay code	No. false positives									Specificity (%) [95% CI**]
	Healthy persons n = 50	Hepatitis B n = 20*	Clonorchiasis n = 20	Paragonimiasis n = 10*	Trichinellosis n = 10	Ascariasis n = 10	Hookworm disease n = 10	Trichuriasis n = 10	Total n = 40*	
IHA_AJ	3	1	2	5	2	0	0	1	14	90.0 [85.0–95.0]
IHA_HB	4	1	2	6	3	0	0	2	18	87.1 [81.6–92.7]
IHA_XY	15	4	10	6	6	0	1	0	42	70.0 [62.4–77.6]
IHA_JX	1	0	1	5	2	0	0	1	10	92.9 [88.6–97.1]
DIGFA_SH	1	0 (7)	1	2 (5)	2	0	0	0	6 (122)	95.1 [91.2–98.9]
DIGFA_XC	0	0 (11)	1	4 (9)	3	1	0	0	9 (130)	93.1 [88.7–97.4]
DIGFA_ZJ	1	1 (13)	0	6 (9)	3	0	1	0	12 (132)	90.9 [86.0–95.8]
MELISA_BE	1	0	1	5	1	0	0	0	8	94.3 [90.5–98.1]
ELISA_SZ	2	1	0	5	1	0	0	0	9	93.6 [89.5–97.6]
EITB	1	0	1	0	2	0	0	0	4	97.1 [94.4–99.9]

*() indicates number of tests that gave valid results;

**CI: confidence interval.

The specificity of each two tests was compared (Table 4). The specificity of IHA_XY was significantly lower than those of other evaluated tests. Compared to the EITB, which had the highest specificity, four tests (IHA_XY, IHA_AJ, IHA_HB, and DIGFA_ZJ) performed with significantly lower specificities (*P* <0.05). The other five tests, which did not differ from the EITB in specificity, also showed no difference between any two tests (*P*>0.05).

**Table 4:** Comparative differences in testing specificity (*P* values).

Assay code	IHA_AJ	IHA_HB	IHA_XY	IHA_JX	DIGFA_SH	DIGFA_XC	DIGFA_ZJ	MELISA_BE	ELISA_SZ	EITB
IHA_AJ										
IHA_HB	0.452									
IHA_XY	<0.001**	<0.001**								
IHA_JX	0.393	0.111	<0.001**							
DIGFA_SH	0.122	0.026*	<0.001**	0.453						
DIGFA_XC	0.365	0.104	<0.001**	0.944	0.501					
DIGFA_ZJ	0.272	0.727	<0.001**	0.053	0.011*	0.051				
MELISA_BE	0.276	0.068*	<0.001**	0.812	0.600	0.871	0.031*			
ELISA_SZ	0.183	0.039*	<0.001**	0.626	0.775	0.683	0.017*	0.802		
EITB	0.026*	0.003*	<0.001**	0.168	0.522	0.157	0.001*	0.255	0.377	

*indicate significant difference;

**indicate highly significant difference.

**Figure 1:**
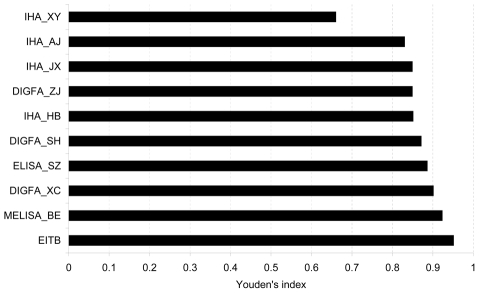
Validity of evaluated tests for the diagnosis of *S. japonicum* infection (Youden's index).

Overall assay performance was evaluated using Youden's indices (Fig. 1). Except for the IHA_XY, all tests demonstrated good performance as indicated by Youden's indices higher than 0.80, especially the MELISA_BE and the EITB (0.92 and 0.95, respectively).

**Figure 2:**
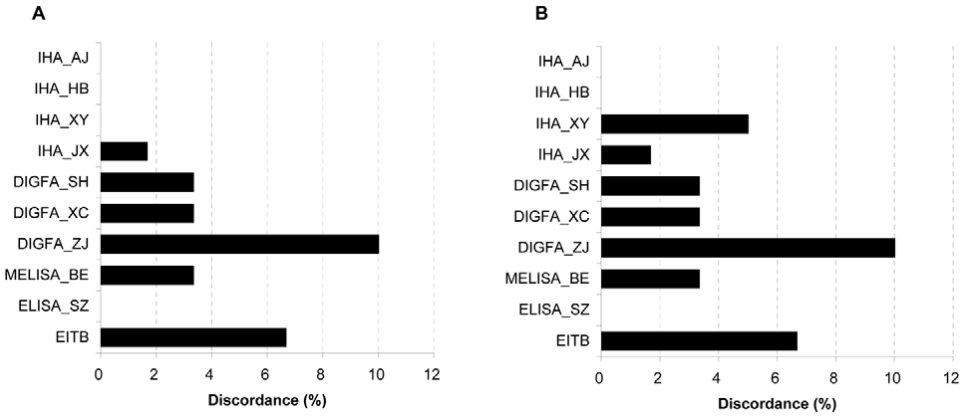
Test reproducibility results (discordance %). A: Operator-to-operator variability (n = 60). B: Run-to-run variability (n = 60).

For all tests, reproducibility was measured by determining operator-to-operator and run-to-run variation. The results are summarized in Fig. 2. Overall, variability was low. The maximum variability observed was 10.0% (6/60) for DIGFA_ZJ test for operator-to-operator and run-to-run variation, respectively, while no variance was detected in the IHA_AJ, IHA_HB, and ELISA_SZ assays. The scores for operational characteristics are summarized in Table 5. Three DIGFA assays obtained the best scores on the questionnaire (10/10) but all presented technical problems with membrane permeability, as evidenced by several serum specimens that could not penetrate. The MELISA_BE received the lowest score (5/10). All tests scored 3 for clarity of kit instructions. Given the similar test principles, four IHA assays and three DIGFA assays were scored equally for technical complexity (2/3, 3/3, accordingly), while MELISA_BE and EITB scored the lowest (1/3). The three DIGFAs scored the highest for ease of interpretation (3/3) and no extra equipment except handheld micropipettes and micropipette tips was needed. The MELISA_BE and ELISA_SZ scored the lowest on ease of interpretation because results were generated using an enzyme-linked analyzer.

**Table 5:** Operational characteristics of the evaluated tests.

Assay code	Clarity of instructions	Technical complexity	Ease of interpretation of results	Equipment required but not supplied	Total score	Technical problem
IHA_AJ	3	2	2	0	7	No
IHA_HB	3	2	2	0	7	No
IHA_XY	3	2	2	0	7	No
IHA_JX	3	2	2	0	7	No
DIGFA_SH	3	3	3	1	10	Yes
DIGFA_XC	3	3	3	1	10	Yes
DIGFA_ZJ	3	3	3	1	10	Yes
MELISA_BE	3	1	1	0	5	No
ELISA_SZ	3	2	1	0	6	No
EITB	3	1	2	0	6	No

For each test, a score of 3 for the first three characteristics was given with 3 being the best, and then a score of 1 was given if the test does not require any additional equipment.

## Discussion

Parasitological techniques, the definitive methods for determining *S. japonicum* infection, are relatively insensitive in populations with light infections, as is the case in P.R. China after years of integrated schistosomiasis control [20], [24], [35]. Other diagnostic alternatives include immunologic methods for detection of parasitic specific antibodies or circulating antigens. Although a number of monoclonal antibodies developed for detection of circulating antigens have been developed in P.R. China, these assays showed unsatisfactory sensitivity, especially in patients with light infections [36]–[38]. Antibody-detection has been shown to be more sensitive than stool examination and is needed in areas characterized by low level of transmission, low prevalence and particularly low intensity [39]–[41]. To identify quality-assured diagnostic assays for use in schistosomiasis control areas with low endemicity, we compared diagnostic tests developed in P.R. China in a controlled laboratory setting using well-characterized archived serum specimens (Figure S1), and prioritized the methods for further assessment in field settings.

Once transmission of the disease drops to very low levels, the positive predictive value for any test is decreased. Therefore a confirmatory method may be needed to verify results obtained in the field, especially in areas of low prevalence. The ideal confirmatory method should be independent of the first test method to increase the statistical reliability of the results. The EITB methods using *S. mansoni* and *S. haematobium* adult worm microsomal fractions have been demonstrated to be a highly sensitive and specific for detection of schistosomiasis caused by these species [34], [42]. The EITB using *S. japonicum* adult worm microsomal fractions was evaluated here to determine if this test would be a suitable reference method to confirm *S. japonicum* infections in especially low transmission settings. The EITB was also chosen for evaluation here because the assay format is independent of the other methods evaluated in this study, all of which detect antibodies to egg antigens. Instead the EITB detects antibodies to adult worm antigens presented in the microsomal fraction and thereby provides an independent means of confirming positives identified using the field tests. In this study, the EITB performed with a high sensitivity for detection of patients with light *S. japonicum* infections and also had a low cross-reaction rate with normal or heterologous sera. Considering these data and other studies, the EITB has been demonstrated to be effective and specific for detection of schistosome infections. For these reasons, we have identified the EITB as a possible method for definitive serodiagnosis of *S. japonicum* infections.

The nine other diagnostic tests evaluated demonstrated a good ability to identify schistosomiasis patients with light infections, with sensitivities in the range of 92.0% (95% CI: 86.7–97.3%) to 98.0% (95% CI: 95.3–100.0%). This observation is consistent with a previous study performed by our laboratory with archived serum specimens, in which most immunoassays had sensitivities above 90% [43]. We also found that the serum specimens that generated the most false negative results were collected from patients with very low infection intensities (generally less than 20 EPG). Thus, more sensitive assays need to be developed to avoid misidentifying those individuals with low level infections, but who pose a tremendous risk for continuing transmission. There are quite a few reports of PCR-based methods for detecting *S. japonicum* infection [14]–[17]. Although the application of molecular techniques in field studies needs further evaluation, these techniques could be improved and standardized as laboratory reference or confirmatory assays because of their excellent sensitivity and specificity.

The specificities of the nine assessed tests in P.R. China varied greatly and ranged from 70.0% (95% CI: 62.4–77.6%) to 95.1% (95% CI: 91.2–98.9%). Four tests, including the IHA_AJ, IHA_HB, IHA_XY, and DIGFA_ZJ, with specificities lower than 91%, differed significantly in specificity from that of the EITB (specificity: 97.1%, 95% CI: 94.4–99.9%). The remaining five assays, IHA_JX, DIGFA_SH, DIGFA_XC, MELISA_BE and ELISA_SZ, did not differ between any two tests in specificity. The specificities of the evaluated tests were mainly influenced by the cross-reactivity with specimens from paragonimiasis or trichinellosis patients. These observations suggested that when a positive result is obtained for a patient from an area co-endemic with schistosomiasis and paragonimiasis or trichinellosis, the history of infection with, or exposure to, the other two diseases should be considered also.

In addition to high sensitivity and specificity, an ideal diagnostic test to be considered for use in developing countries should be user-friendly, rapid and robust, require simple or no equipment, be deliverable to end-users, and be manufactured according to quality standards [44], [45]. First, reproducibility, which is a measure of the closeness of agreement between test results when the conditions for testing or measurement change [46], is an intrinsic factor that may affect performance especially when used in the field for a large-scale evaluation. Our study measured the reproducibility of assays by determining operator-to-operator and run-to-run variation. The discordant rate in the range of 0-10.0% indicates that all tests performed with good reproducibility. Second, the operational characteristics of each test were assessed based on a questionnaire administered to technicians performing the assays. Results from the questionnaire indicated that three DIGFA tests had the greatest advantage for field use, because of their short time to test completion, no extra equipment requirements and ease of interpreting results. We did note that membrane permeability needs to be improved in the DIGFA assays. Further modification for the DIGFA_SH and DIGFA_XC methods is necessary because the specimen volumes needed requires collection of whole blood via venipuncture. This is a limitation because well trained personnel are required and the practice is not always widely accepted in all populations. A later assessment of modified DIGFA tests, which only required a 25 µl serum specimen showed improved membrane permeability; however, only the DIGFA_SH still had high sensitivity and specificity, while the sensitivity of the other two DIGFA tests had decreased below 90% (data not shown). The IHA tests are the most widely used assays in P.R. China and no technical problems with these were identified except the need for an incubator. The ELISA_SZ, which had a high sensitivity and specificity and could detect specimens in panels and give quantitative results based on OD values, is more suitable for a large-scale community survey. However, the needs for a 37°C incubator and a microplate reader for results interpretation limit its use to settings with some moderate amount of laboratory resources. The MELISA_SE, which performed with highest sensitivity and specificity, similar to the EITB, is more appropriate for use as a reference laboratory method or a clinical diagnostic assay because of its complicated procedures and instrument requirements. If it is to be used in field trials, its operational procedures still need simplifying.

The specimen type for all of the tests evaluated here was serum, which means the whole blood collected from donors should be centrifuged or kept at a low temperature for a long time to promote clot formation. The use of whole blood or dried whole blood could negate the difficulties of processing and transporting blood samples for serologic surveys in rural areas of P.R. China.

In conclusion, most immunodiagnostic tests evaluated had an acceptable level of performance relative to the ‘gold’ standard Kato-Katz technique. After considering all the requirements of field diagnostics, i.e., sensitivity, specificity, validity, and ease of use, the IHA_JX, DIGFA_SH, and ELISA_SZ were selected for further field trials. With the caveat that the diagnostic approaches may need to be adjusted to the stage of control and the objective of the control activity [43], we expect to prioritize those tools depending on the specific need [47], [48], such as screening chemotherapy targets, assessing the efficacy of schistosomiasis control programs or monitoring the endemic status of schistosomiasis.

## Supporting Information

Alternative Language Abstract S1 Translation of the Abstract into Chinese by Jing Xu.
(0.02 MB DOC)

Figure S1 Flowchart used for studies of diagnostic tests.
(2.18 MB TIF)
