# Supplementary material for: Evaluation of Immunoassays for the Diagnosis of Schistosoma japonicum Infection Using Archived Sera
Source: PLoS Negl Trop Dis. 2011 Jan 18;5(1):e949. doi: 10.1371/journal.pntd.0000949 (PMC3022531; doi:10.1371/journal.pntd.0000949)
Supplement: Alternative Language Abstract S1 — Translation of the Abstract into Chinese by Jing Xu. (0.02 MB DOC) [file pntd.0000949.s001.doc]

**用存档血清评估九种免疫试验诊断日本血吸虫感染的效果**

许静^1^，Rosanna W. Peeling^2^, 陈家旭^1^，吴晓华^1^，吴忠道^3^，汪世平^4^，冯婷^1^，陈绍红^1^，李浩^1^，郭家钢^1^，周晓农^1^**^*^**

1 中国疾病预防控制中心寄生虫病预防控制所，上海，中国，2 伦敦卫生和热带医学院诊断研究室，伦敦，英国， 3 中山大学中山基础医学院，广州，广东，中国 4 中南大学湘雅医学院，长沙，湖南，中国

*Corresponding author: E-mail: ipdzhouxn@sh163.net

*通讯作者：E-mail: ipdzhouxn@sh163.net

**摘要**

***背景：***随着旨在降低中国血吸虫病传播的国家项目的启动，血吸虫病低度流行区迫切需要质量有保证的诊断试剂，以用于病例检测、化疗及其他血吸虫病控制干预措施效果的监测。我们比较了在中国研制的九种用于血吸虫病抗体检测的免疫诊断试剂的性能，并分析了他们进一步开展现场评估的优先性。

***方法学/主要发现***：以Kato-Katz法为参考标准，中国研制的9种免疫诊断试剂分别检测240份标记完好的归档血清样本（100份阳性，140份阴性）。另外，美国疾控中心提供的酶联免疫电转印迹试验（EITB）也参与了测评。除敏感性和特异性外，每种试剂的重复性通过不同操作者、不同操作次数的变异进行了评估。另外，还对每种试剂终端用户操作的简易性进行了评估。各免疫诊断试剂均具有较高的敏感度（92.0% (95%CI: 86.7-97.3%)- 98.0% (95%CI: 95.3-100%)），特异度为70.0% (95%CI: 62.4-77.6%) - 97.1% (95%CI: 94.4-99.9%)。所有试剂均表现出很好的重复性，不同操作者、不同操作次数的不一致率范围在0-10%。除一种基于磁珠颗粒的酶联免疫吸附试验外，其他试剂均简单易用，尤其是金标免疫渗滤试验。

***结论/意义：*** 大多数评估的试剂均具有较好的操作性能并能对血吸虫病防治产生影响。三种具有较高的敏感性、特异性且易于使用的试剂可用于下一步的现场评估。
