# Supplementary figures and images for: Evaluation of Immunoassays for the Diagnosis of Schistosoma japonicum Infection Using Archived Sera
Source: PLoS Negl Trop Dis. 2011 Jan 18;5(1):e949. doi: 10.1371/journal.pntd.0000949 (PMC3022531; doi:10.1371/journal.pntd.0000949)

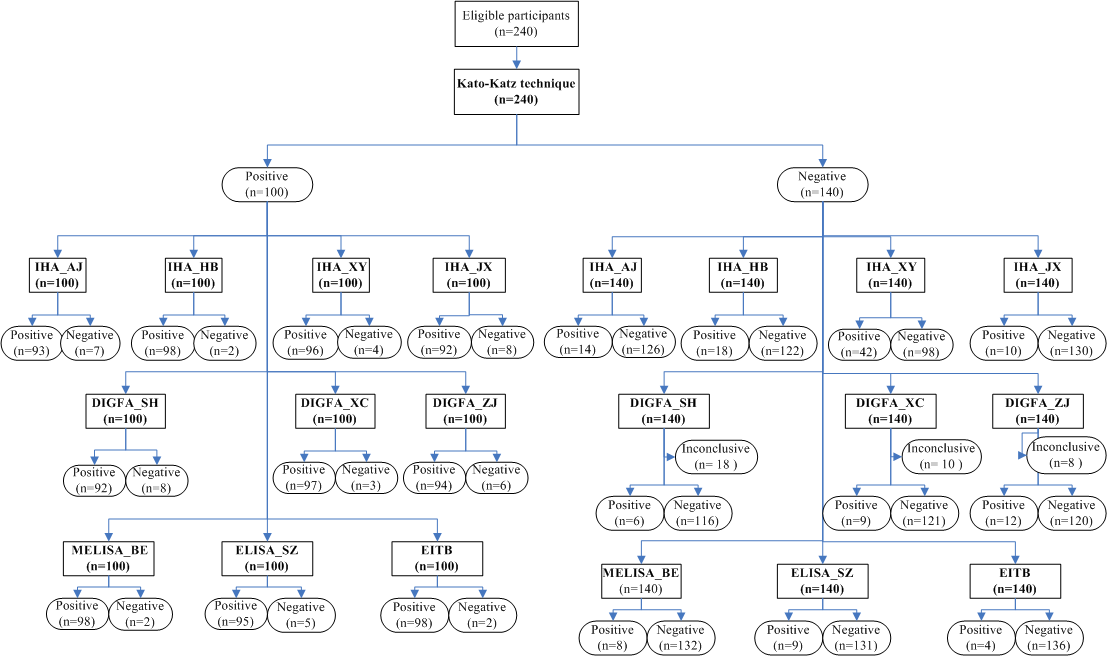

Supplement: Figure S1 — Flowchart used for studies of diagnostic tests. (2.18 MB TIF) [file pntd.0000949.s002.tif]
